# Comparison of Gut Microbiota in Two Different Maternal Exposure Models of Autism Spectrum Disorder in Mice

**DOI:** 10.31083/AP38790

**Published:** 2025-02-28

**Authors:** Qiang Zhang, Xuying Pang, Min Guo, Yuezhu Wang, Yu Xu, Quan Li, Huajun Zheng

**Affiliations:** ^1^Department of Obstetrics and Gynecology, Affiliated Hospital of Zunyi Medical University, 563000 Zunyi, Guizhou, China; ^2^Shanghai-MOST Key Laboratory of Health and Disease Genomics, NHC Key Lab of Reproduction Regulation, Shanghai Institute for Biomedical and Pharmaceutical Technologies, Fudan University, 200032 Shanghai, China

**Keywords:** autism spectrum disorder (ASD), chemical exposure, mouse model, gut microbiota

## Abstract

**Background:**

Autism spectrum disorder (ASD) is a group of neurodevelopmental disorders with unknown etiology and unclear pathogenesis. Although construction of animal models of ASD using chemical exposure during pregnancy is a mature technique, the gut microbiota of these exposure models induced using different chemicals in mice have not been compared.

**Methods:**

To compare the effects of exposure to different chemicals during pregnancy on the composition of gut microbiota in offspring, we treated Institute of Cancer Research (ICR) mice with lipopolysaccharide (LPS) and valproic acid (VPA) during pregnancy to construct different offspring ASD mouse models. After successful model construction, the gut microbiota of these models were studied.

**Results:**

After adjusting for the random effects of the litter, the two groups showed a significant reduction in social time (social deficits) and an increase in self-grooming behaviors (repetitive and stereotyped behaviors). Gut microbiota analysis revealed significant changes, mostly a decrease, in the abundance of four phyla, 52 genera, and 41 species in the two types of ASD models. Several different gut microbes could be related to the development of ASD.

**Conclusions:**

Chemicals exposure during pregnancy induces ASD-related behavioral abnormalities in offspring mice. Importantly, exposure to different chemicals during pregnancy produces varying degrees of effects on gut microbiota composition in offspring ASD models. This finding can provide a reference for studies on the etiology and pathogenesis of ASD.

## Main Points

1. Exposure to different chemicals during pregnancy can induce varying degrees of autism spectrum disorder (ASD)-like behaviors in offspring mice.

2. Exposure to different chemicals during pregnancy was able to affect gut microbiota in offspring ASD mice.

3. Changes in the gut microbiota of ASD mouse models may be closely related to different autism-like behaviors.

## 1. Introduction

Autism spectrum disorder (ASD) is a group of complex neurodevelopmental disorders, characterized by language development disorder, social interaction defects, and repetitive and stereotyped behaviors. ASD is accompanied by one or more neurodevelopmental symptoms, such as attention-deficit/hyperactivity disorder, self-mutilation, specific learning disorder, and memory disorder [1, 2]. The global prevalence of ASD is approximately 0.6%, but there are some differences in different countries or regions [3], with the incidence in male children being 4–5 times higher than in females [4]. Among individuals with ASD, only a few can take care of themselves, while most often need family and social care. Furthermore, there is no known cure for ASD [5] and it seriously affects the quality of life of patients, imposing a heavy burden on their families and society [6]. Therefore, understanding the etiology and pathological mechanisms of ASD is crucial for the rehabilitation of patients with ASD.

However, the etiology and pathogenesis of ASD remain unclear, although it might be the result of interactions between genetics and environment [7, 8]. Accumulating research evidence has shown that mutations in *KDM5B* [9] or *TRAPPC9* [10], and deletion of *PTEN* [11], as well as polymorphism in the transcription factor *ZNF804A* [12] may lead to ASD. These factors have also led to the development of the ASD mouse model. Rodent models provide a valuable tool to understand the causal role of genetic and environmental factors that lead to ASD [13]. There are more than 20 mouse models of ASD, obtained through gene knockout or mutation [14] or from various inbred mice, including the BTBR and C58/J models that exhibit autism-like behaviors [13]. However, genetic factors can only explain some ASD cases [15]. Among the numerous gene mutations and copy number variants associated with autism, each variant is present in only a few individuals with ASD. Thus, mouse models based on genetic modification of ASD-related mutations represent only a few cases. Increasing evidence suggests that maternal exposure during pregnancy to high-risk environmental factors, including valproic acid (VPA) [16, 17], and immune activation (infection) [18] significantly increases the risk of ASD in offspring.

VPA is a short-chain fatty acid (SCFA) and an antiepileptic drug and mood stabilizer. Mothers using VPA during early pregnancy have an increased risk of ASD in their offspring [16, 17]. Sprague–Dawley rats injected with VPA on day 12 of pregnancy produced offspring with reduced sociability similar to human autistic behavior [19]. The offspring of Institute of Cancer Research (ICR) mice exposed to VPA on day 12.5 of pregnancy had social interaction defects, learning deficits, anxiety-like behavior, and increased neocortical neuron density, similar to the phenomena in human ASD [20].

Maternal immune activation (MIA) caused by infection during pregnancy is a high-risk factor for neurodevelopmental disorders in offspring [21]. Lipopolysaccharide (LPS), the cell wall component of gram-negative bacteria, is generally administered to animals during pregnancy to construct an MIA model. A single intraperitoneal injection of LPS on day 9.5 of pregnancy led to rat offspring developing communication and socialization defects similar to ASD and increased repetitive and restrictive behaviors [22, 23].

Patients with ASD exhibit varying degrees of gastrointestinal symptoms [24, 25]. The gut microbiota may be involved in the pathogenesis of ASD [26]. The “microbe–gut–brain axis” is a bidirectional communication pathway between the gut microbiome and central nervous system [27], which plays a crucial role in the interaction between the gut microbiota and the brain [28, 29]. Several metabolites, including SCFAs, 5-hydroxytryptamine (5-HT), and gamma-aminobutyric acid (GABA) produced by the gut microbiota, can enter the blood owing to increased permeability of the intestinal mucosal barrier and subsequently enter the brain through the blood–brain barrier (BBB) or vagus nerve pathway, affecting brain function and leading to abnormal behaviors and language development disorders [29, 30]. Thus, alterations in the composition of the gut microbiota might participate in ASD pathogenesis by affecting the host’s physiological functions, immunological system, material metabolism, neuroendocrine, and other functions [28, 30].

Although LPS and VPA are used for the construction of ASD models, the two models have not been compared. Herein, these two chemicals were used to construct ASD progeny mouse models that were used to analyze their gut microbiota.

## 2. Materials and Methods 

### 2.1 Animals

ICR mice (5–8 weeks old, SPF) purchased from Shanghai Jiesijie Experimental Animal Co., Ltd. (certificate number: 20180004022141/20180004023836, Shanghai, China) were reared in the Barrier Environment Rodent Room of the Shanghai Institute for Biomedical and Pharmaceutical Technologies. The mice were kept at 22 ± 2 °C under 30%–70% relative humidity and 12-hour light/dark cycles and had ad libitum access to food (lab irradiation sterilized rodent diet) and water (high-pressure sterilized tap water). Animal breeding and experiments were approved by the Experimental Animal Ethics Committee of the Shanghai Institute for Biomedical and Pharmaceutical Technologies (former Shanghai Institute of Planned Parenthood Research) (approval number: 2019-21, date: March 5, 2019).

### 2.2 Grouping and Administration of Chemicals to Pregnant Mice

The ICR mice (8–10 weeks old) were mated at night and females were examined every morning. The day when vaginal plugs were observed was considered gestation day 0.5 (GD 0.5). Twenty-two pregnant mice were randomly divided into three groups: VPA (n = 8), LPS (n = 8), and Control (n = 6).

On GD 12.5, pregnant mice in the LPS and VPA groups received a single dose of LPS (100 µg/kg, Shanghai Yuanye Bio-Technology Co., Ltd, batch number: S10M11I112569, Shanghai, China) [20] or VPA (500 mg/kg, Shanghai Yuanye Bio-Technology Co., Ltd, batch number: Y18J7C16394, Shanghai, China) [22], respectively, by intraperitoneal injection, whereas the Control group was treated with the same volume of saline (Shanghai Yuanye Bio-Technology Co., Ltd, batch number: L21D11G135094, Shanghai, China). The offspring mice were segregated into cages (4–5 per cage) according to sex at postnatal day 21.

### 2.3 Behavior Testing of the Offspring

At 6–8 weeks after the birth of the offspring, behavioral testing was performed using open field and three-chamber sociability tests. The interval between the two tests was 3–4 days to allow sufficient rest time for the mice. The test time was 8:00 am–8:00 pm. The mice were moved to the test room and adapted to the environment for at least 30 minutes [31].

#### 2.3.1 Open Field Test (OFT)

The OFT was carried out in a dark blue box (50 cm × 50 cm × 40 cm) which was made of polyvinyl chloride (PVC) board (nonporous), with a black bottom plate and no top cover. The mice were gently placed into the central area (25 cm × 25 cm) of the open field and were left to explore freely for 10 minutes. They were then tested for 10 minutes. Before and after the test, 75% ethanol was used to sterilize the bottom and surrounding baffles of the arena. An overhead camera was used for recording. The VisuTrack animal behavior analysis software (version 3.0, Shanghai Xinruan Information Technology Co., Ltd., Shanghai, China) was used for tracking and analysis.

#### 2.3.2 Three-chamber Sociability Test

The three-chamber sociability test was performed in a transparent PVC box (60 cm × 40 cm × 22 cm) divided into three chambers (20 cm × 40 cm × 22 cm) with two clear PVC dividers. After 10 minutes of adaption, the mice were confined to the center chamber and a stimulus mouse (sex-matched ICR mouse not raised in the same cage) was placed in a restraint cage in one chamber (social chamber), whereas a white object, almost the same size as a mouse, was placed in a restraint cage in the other chamber (non-social chamber). The mice were able to travel freely within the three chambers for 10 minutes, and their movements were recorded with an overhead camera and tracked using the VisuTrack software.

### 2.4 Statistical Analysis

Data were analyzed using R software (version 4.1.2, University of Auckland, Auckland, New Zealand, https://www.r-project.org/). Welch’s *t*-test was used for the statistical analysis of two sets of independent samples with unequal population variance. The package “lmerTest” was used to construct mixed linear models with treatment factors LPS and VPA as fixed effects and litters as random effects according to litter numbers.

The package “lme4” was used to judge whether the random effect was significant. Differences in random effects were compared using the Log Likelihood Ratio Test (LRT) obtained by Restricted Maximum Likelihood (REML), an analysis designed to test whether the variance between litters is zero. The Intraclass Correlation Coefficient (ICC) was calculated to determine the aggregation of mice in the same litter. The *t*-test with Satterthwaite’s method was used for the fixed factor. The OriginPro (version 2019b, OriginLab Corporation, Northampton, MA, USA.) analysis software was used to perform the Mann-Whitney test on the measured behavioral data to determine various models and the control group. Mean ± standard deviation was used to describe the data with normal distribution, and median (IQR) was used to describe the data with skewed distribution. The level of significance was set at α = 0.05.

### 2.5 Fecal Sample Collection and MiSeq of ASD Mouse Models

#### 2.5.1 Fecal Sample Collection of Offspring Mice 

At the sixth week after the birth of the offspring, the feces of each male mouse were collected. Collection time was 2:00 pm–4:00 pm; each mouse was placed in a clean box alone and 4–5 grains of feces were collected for each mouse. The diet of the mice was not restricted before collection, but mice were fasted during collection. After fecal collection, the mouse was returned to the original cage. The fecal sample was stored at –80 °C until DNA extraction.

#### 2.5.2 Genomic DNA Extraction, PCR Amplification, and 16S Ribosomal RNA (16S rRNA) Gene Sequencing

DNA extraction and PCR amplification were performed as described previously [32]. Fecal DNA was extracted using the QIAamp DNA Stool Mini Kit (QIAGEN, Hilden, Germany). The V3-V4 region of 16S rRNA genes was amplified using primers 338F and 806R [33] with TransStart FastPfu DNA Polymerase (TransGen, Beijing, China) in 20 cycles. Amplicons from three replicate PCRs of each sample were purified using the AxyPrep DNA Gel Extraction kit (AXYGEN, Union City, CA, USA), and pooled at equal concentrations after quantification. Next, 2 × 300 paired-end sequencing was performed for the equivalent pooled 16S rRNA PCR amplicons on an Illumina MiSeq instrument (San Diego, CA, USA).

#### 2.5.3 Bioinformatics and Statistical Analysis

Sequencing data were analyzed using Mothur (version 1.39.5, The University of Michigan, Ann Arbor, Michigan, USA) [34] as described previously [32]. Reads containing ambiguous bases, that were shorter than 350 base pairs, or that had chimeric or contaminant sequences, were first removed. Thereafter, the SILVA reference database (version 132) [35] was used as a reference for the identification of operational taxonomic units (OTU) under a threshold of 97% similarity. Community richness, evenness, and diversity were assessed using Mothur. The taxonomic assignments were based on the Ribosomal Database Project (RDP) [36] with the default parameter (80% threshold). The RDP classifier [37] Bayesian algorithm was used to perform taxonomic analysis on the representative sequences of OTUs. Microbiota functions were predicted using the phylogenetic investigation of communities by reconstruction of unobserved states (PICRUSt) (version 1.1.0, Dalhousie University, Nova Scotia, Canada) [38]. The bacterial differences among the three groups were assessed using permutational multivariate analysis of variance (PERMANOVA) in the PAST4 software package (version 4.16c, University of Oslo, Oslo, Norway), with default parameters based on Bray-Curtis distance [39]. Significant differences in the relative abundance of microbial taxa (phylum, genus, and species) and microbiota functional profiles between the ASD and control groups were analyzed using the two-sided White’s non-parametric *t*-test in the STAMP (version 2.1.3, Dalhousie University, Nova Scotia, Canada) software package [40].

## 3. Results

Spontaneous abortion occurred before drug intervention in two pregnant mice in the VPA group and two in the Control group, and vaginal bleeding and miscarriage occurred in four pregnant mice of the LPS group within 24 to 48 hours after injection. Finally, 14 pregnant mice delivered successfully, and 158 offspring (75 males, 83 females) were born (**Supplementary Table 1**). As age increased, the mean weight of the male offspring was significantly higher than that of the female offspring (**Supplementary Table 2**). Furthermore, as ASD-like behaviors in mice are sex-dependent [41], we only performed behavioral tests and gut microbiota studies in the 75 male mice.

### 3.1 Behavioral Analysis of Offspring Mice

**Fig. 1.**
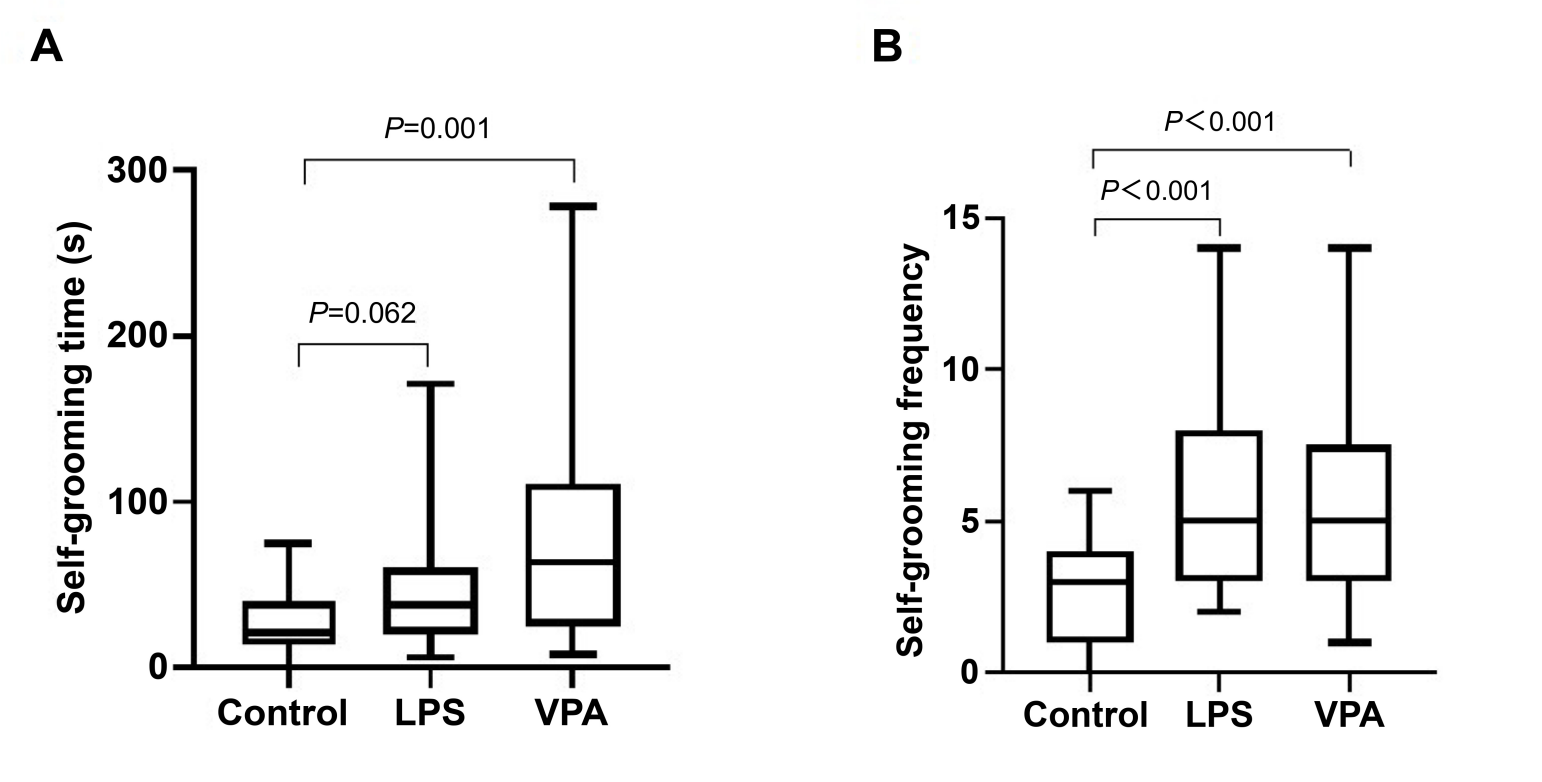
**Box-plot distribution of self-grooming time (A) and frequency (B) of mice in different groups**. Comparisons between groups (Control vs LPS and Control vs VPA) were performed using the Mann-Whitney U test. LPS, lipopolysaccharide; VPA, valproic acid.

The behaviors of self-grooming and sociability were analyzed, and random effects analysis was used to test the litter effects. In the open field test, the results showed that self-grooming time (adjusted ICC = 0.479) and frequency (adjusted ICC = 0.353) of the LPS group were affected by litter effects (**Supplementary Tables 3,4**). After controlling for the random effects of the litters, the self-grooming time of mice in the VPA group, and the self-grooming frequency of both the VPA and LPS, groups were significantly increased compared with the Control group (Fig. 1, **Supplementary Table 5**).

**Fig. 2.**
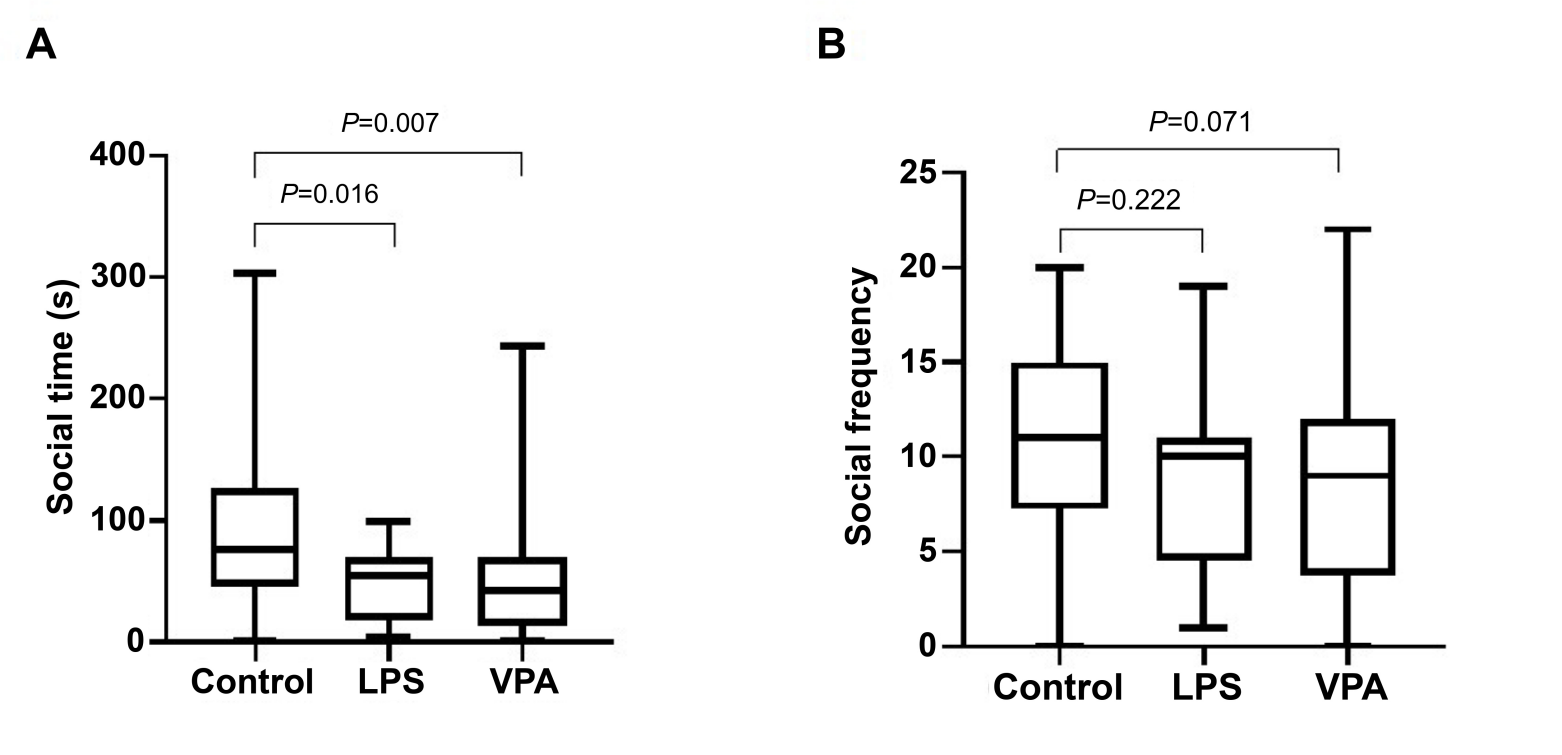
**Box-plot distribution of social time (A) and social frequency (B) of mice in different groups**. Comparisons between groups (Control vs LPS and Control vs VPA) were performed using the Mann-Whitney U test.

In the three-chamber sociability test, the Control group mice spent significantly more time in the social chamber than the two model groups. Although the social frequencies were decreased in the two model groups, there were no significant differences relative to the Control group (Fig. 2). This suggests the presence of social deficits in the two ASD models.

### 3.2 Analysis of the Gut Microbiota

**Fig. 3.**
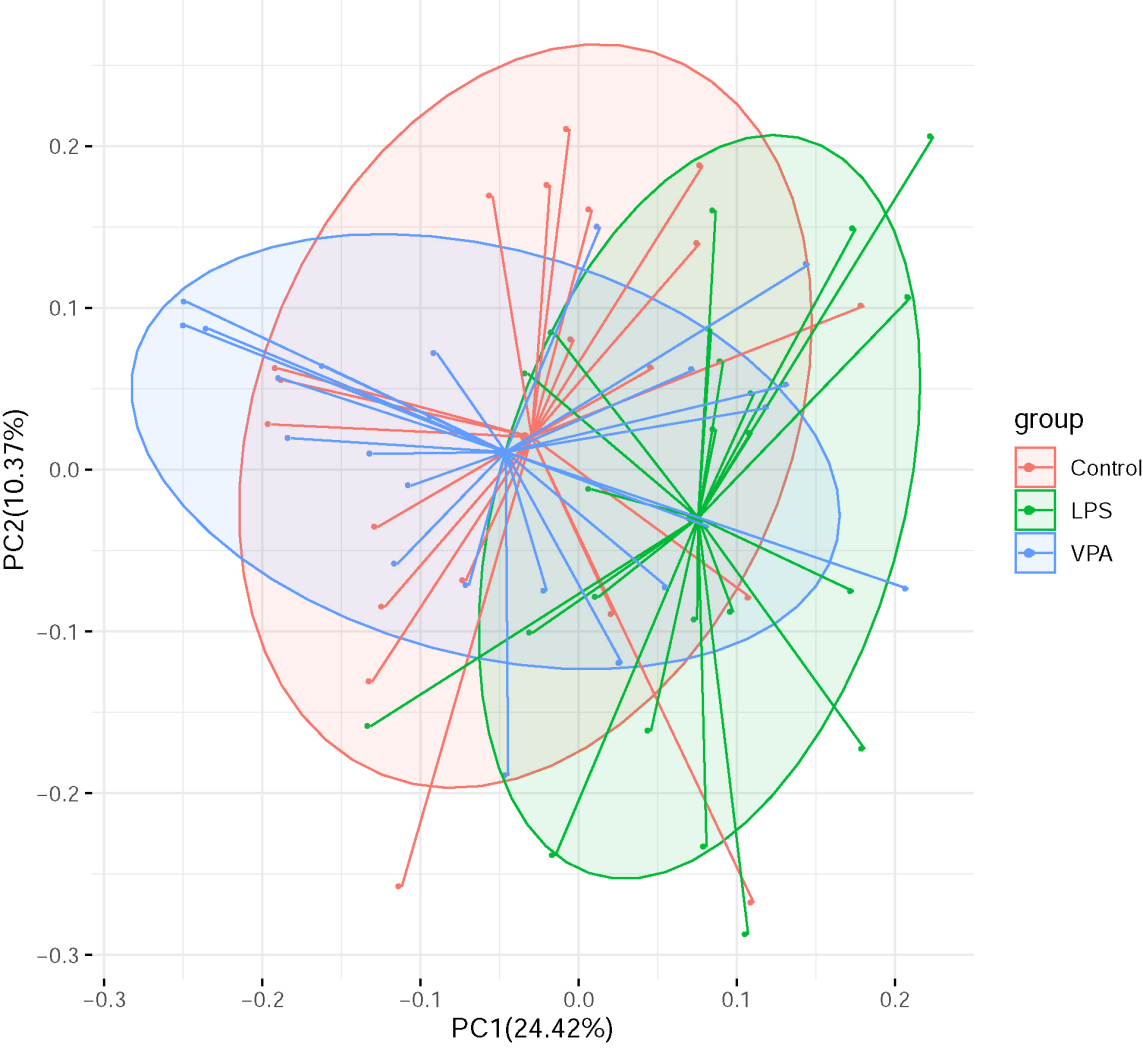
**PCA calculated by weighted UniFrac distances**. The *p* value from the PERMANOVA analysis of each ASD model group and the Control group was less than 0.001. PCA, principal component analysis; ASD, autism spectrum disorder.

We collected 75 fecal samples from the male mice (21 in the LPS group, 30 in the VPA group, and 24 in the Control), which were used for analysis of the gut microbiota. A total of 2,492,284 (21,951–48,782) high-quality 16S rRNA gene sequences were obtained from the 75 samples. To standardize the data and avoid statistical bias, 20,299 16S rRNA gene sequences were randomly selected from each sample to analyze the bacterial community structure, and to calculate the abundance, uniformity, and diversity of the gut microbiota. A total of 674 OTUs were obtained from the sequences of 75 samples and classified into 10 phyla, 87 genera, and 90 species. Good’s coverage for the two ASD models and Control group exceeded 99.9% (Table 1). The Shannon index for the LPS model was lower than that of the Control group, whereas that for the VPA model was higher (Table 1). Moreover, the principal component analysis (PCA) showed that the distance between the Control group and the VPA group was closer (Fig. 3), although significant differences were found between each ASD model group and the Control group using PERMANOVA analysis (*p* < 0.001).

**Table 1.** **Evaluation of gut microbiota diversity in the three groups**.

Group	Sample	OTUs	Coverage	Richness		Evenness	Diversity
				Chao	ACE	Simpsoneven	Shannon
Control	24	432	0.99995	440.05	440.33	0.04281	4.03
LPS	21	386	0.99996	391.83	391.82	0.02355	3.41
VPA	30	465	0.99996	472.29	472.24	0.04139	4.05

Note: Good’s coverage—if the value is greater than 90%, the sequencing volume is sufficient for analysis. LPS, lipopolysaccharide; VPA, valproic acid; OTUs, operational taxonomic units; ACE, abundance-based coverage estimator.

Compared with the Control group, there were significant differences in three phyla in the LPS model (*Candidatus Saccharibacteria*, *Proteobacteria*, and *Verrucomicrobia*), whereas there was only one phylum (*Campilobacterota*) statistically different in the VPA model group (Table 2).

**Table 2.** **Significantly different phylum between ASD models and the Control group**.

Phylum	Control			LPS				VPA			
	Min (%)	Max (%)	Median (%)	Min (%)	Max (%)	Median (%)	*p* value	Min (%)	Max (%)	Median (%)	*p* value
*Bacteroidetes*	14.917	55.535	29.171	5.660	51.451	33.159	0.661	14.474	80.452	39.460	0.231
*Campilobacterota*	0.296	11.203	1.441	0.562	7.345	1.320	0.289	0.222	11.804	3.515	0.025
*Candidatus Saccharibacteria*	0.581	8.247	3.609	0.310	2.581	1.503	0.001	0.143	8.439	2.402	0.605
*Firmicutes*	25.927	77.728	56.057	17.961	77.881	59.826	0.217	10.848	75.073	40.955	0.106
*Proteobacteria*	0.340	12.897	4.572	0.182	4.311	1.084	0.001	0.202	8.848	1.606	0.098
*Verrucomicrobia*	0.000	5.261	0.012	0.000	1.507	0.000	0.020	0.000	4.251	0.000	0.070

Note: Comparisons between groups (Control vs LPS and Control vs VPA) were performed using the two-sided White’s non-parametric *t*-test.

At the genus level, there were 52 significantly different genera in the two ASD models (38 in the LPS group and 24 in the VPA group) compared with the Control group (Table 3). Eight genera, such as *Desulfovibrio*, were significantly decreased in both ASD models, whereas three genera, including *Lachnospira*, *Sporobacter *and *Turicibacter*, were significantly increased in both models. Indeed, in all the 38 genera significantly changed in the LPS model, only four genera, including *Allobaculum*,* Lactobacillus*, *Enterococcus*, and *Vampirovibrio*, were significantly enriched. In the VPA model, nine genera, including *Helicobacter* and *Duncaniella*, were significantly enriched (Table 3).

**Table 3.** **Significantly different genus between ASD models and the Control group**.

Genus	Control			LPS				VPA			
	Min (%)	Max (%)	Median (%)	Min (%)	Max (%)	Median (%)	*p* value	Min (%)	Max (%)	Median (%)	*p* value
*Acetatifactor*	0.000	0.414	0.032	0.000	0.030	0.000	0.002	0.000	0.054	0.000	0.031
*Acutalibacter*	0.000	1.355	0.010	0.000	0.133	0.000	0.038	0.000	0.212	0.025	0.689
*Aerococcus*	0.000	0.414	0.015	0.000	0.000	0.000	0.001	0.000	0.025	0.000	0.001
*Akkermansia*	0.000	5.261	0.012	0.000	1.507	0.000	0.017	0.000	4.251	0.000	0.066
*Alistipes*	0.675	7.050	3.714	0.222	6.769	1.576	0.004	0.399	12.173	2.192	0.862
*Allobaculum*	0.000	0.000	0.000	0.000	5.242	0.000	0.048	0.000	0.000	0.000	1.000
*Amedibacillus*	0.049	1.838	0.268	0.000	0.596	0.163	0.001	0.000	1.227	0.143	0.233
*Anaerofustis*	0.000	0.015	0.000	0.000	0.010	0.000	0.434	0.000	0.005	0.000	0.047
*Anaeroplasma*	0.000	0.034	0.000	0.000	0.054	0.000	0.101	0.000	0.286	0.000	0.009
*Anaerotaenia*	0.000	2.182	0.313	0.000	0.872	0.133	0.002	0.000	1.350	0.202	0.237
*Anaerotruncus*	0.000	0.059	0.000	0.000	0.030	0.000	0.180	0.000	0.148	0.000	0.016
*Burkholderia*	0.000	0.084	0.025	0.000	0.069	0.015	0.009	0.000	0.059	0.012	0.073
*Butyricicoccus*	0.000	0.054	0.000	0.000	0.010	0.000	0.022	0.000	0.020	0.000	0.286
*Butyricimonas*	0.000	0.049	0.000	0.000	0.049	0.010	0.178	0.000	0.128	0.015	0.002
*Christensenella*	0.000	0.064	0.000	0.000	0.059	0.000	<0.001	0.000	0.015	0.000	0.024
*Desulfovibrio*	0.133	4.345	1.131	0.000	0.946	0.074	0.001	0.000	2.015	0.187	0.021
*Duncaniella*	1.015	10.966	2.887	0.621	9.286	4.000	0.617	1.015	10.754	6.111	0.023
*Enterococcus*	0.000	0.025	0.000	0.000	0.054	0.000	0.035	0.000	0.044	0.000	0.486
*Faecalicatena*	0.000	0.103	0.007	0.000	0.069	0.000	0.047	0.000	0.153	0.010	0.724
*Flintibacter*	0.000	1.384	0.434	0.000	0.394	0.118	0.001	0.000	2.365	0.256	0.909
*Harryflintia*	0.000	0.128	0.015	0.000	0.148	0.030	0.444	0.000	0.438	0.039	0.017
*Helicobacter*	0.296	11.203	1.441	0.562	7.345	1.320	0.305	0.222	11.804	3.515	0.029
*Ihubacter*	0.000	0.325	0.069	0.000	0.212	0.039	0.009	0.000	0.158	0.034	0.170
*Intestinimonas*	0.000	0.517	0.143	0.000	0.222	0.030	0.003	0.000	0.833	0.099	0.977
*Jeotgalicoccus*	0.000	0.163	0.000	0.000	0.000	0.000	0.001	0.000	0.000	0.000	0.003
*Kineothrix*	0.015	3.074	0.773	0.000	0.926	0.222	0.001	0.000	2.054	0.219	0.008
*Lachnoclostridium*	0.000	4.384	0.532	0.089	0.665	0.241	0.004	0.025	1.567	0.377	0.370
*Lachnospira*	0.000	0.000	0.000	0.000	0.020	0.000	0.032	0.000	0.030	0.000	0.003
*Lacrimispora*	0.059	8.286	1.271	0.000	1.025	0.089	0.001	0.000	2.453	0.325	0.104
*Lactobacillus*	2.291	51.017	25.381	4.320	67.757	33.342	0.007	0.192	59.929	16.269	0.893
*Lactococcus*	0.000	0.015	0.000	0.000	0.000	0.000	0.032	0.000	0.000	0.000	0.001
*Mailhella*	0.074	7.789	2.404	0.020	1.394	0.394	0.001	0.015	4.990	0.739	0.194
*Monoglobus*	0.000	0.172	0.030	0.000	0.177	0.044	0.950	0.000	0.103	0.015	0.026
*Muribaculum*	0.000	0.030	0.000	0.000	0.079	0.000	0.087	0.000	3.251	0.015	0.008
*Muricomes*	0.000	0.025	0.000	0.000	0.000	0.000	0.025	0.000	0.015	0.000	0.486
*Mycoplasma*	0.000	0.054	0.000	0.000	0.039	0.000	0.271	0.000	0.000	0.000	0.003
*Neglecta*	0.000	1.094	0.224	0.000	0.320	0.020	0.001	0.000	0.897	0.094	0.935
*Odoribacter*	0.202	2.719	1.037	0.059	1.335	0.631	0.008	0.094	3.897	0.897	0.457
*Oscillibacter*	0.000	0.291	0.049	0.000	0.232	0.010	0.001	0.000	0.837	0.037	0.177
*Paludicola*	0.000	0.133	0.034	0.000	0.069	0.010	0.028	0.000	0.241	0.022	0.193
*Phocaeicola*	0.000	0.670	0.121	0.000	0.227	0.034	0.114	0.000	1.754	0.293	0.007
*Pseudoflavonifractor*	0.000	1.547	0.335	0.000	0.340	0.034	0.001	0.000	2.128	0.074	0.310
*Ralstonia*	0.000	0.025	0.000	0.000	0.000	0.000	0.006	0.000	0.000	0.000	0.092
*Roseburia*	0.000	7.818	1.062	0.000	1.685	0.118	0.004	0.000	7.592	0.197	0.329
*Ruminococcaceae incertae sedis*	0.000	0.350	0.049	0.000	0.128	0.034	0.006	0.000	0.236	0.057	0.366
*Ruminococcus*	0.000	0.133	0.005	0.000	0.734	0.020	0.105	0.000	0.369	0.027	0.045
*Saccharibacteria_genera_incertae_sedis*	0.581	8.247	3.609	0.310	2.581	1.503	0.001	0.143	8.439	2.402	0.598
*Sporobacter*	0.000	0.000	0.000	0.000	0.015	0.000	0.252	0.000	0.039	0.000	0.009
*Staphylococcus*	0.000	0.163	0.000	0.000	0.000	0.000	0.001	0.000	0.020	0.000	0.002
*Streptococcus*	0.000	0.138	0.022	0.000	0.079	0.010	0.057	0.000	0.034	0.000	0.007
*Turicibacter*	0.000	0.005	0.000	0.000	19.572	0.064	0.001	0.000	23.105	0.000	0.023
*Vampirovibrio*	0.000	0.015	0.000	0.000	0.034	0.000	0.005	0.000	0.015	0.000	0.238

Note: Comparisons between groups (Control vs LPS and Control vs VPA) were performed using the two-sided White’s non-parametric *t*-test.

At the species level, 41 species with significant differences in the two ASD models were observed (33 in the LPS group and 16 in the VPA group), with most of them being decreased relative to the Control group (Table 4). Notably, the abundance of *Akkermansia muciniphila* was reduced from 1.02% in the Control group to 0.12% in the LPS group and 0.24% in the VPA group.

**Table 4.** **Significantly different species between ASD models and the Control group**.

Species	Control			LPS				VPA			
	Min (%)	Max (%)	Median (%)	Min (%)	Max (%)	Median (%)	*p* value	Min (%)	Max (%)	Median (%)	*p* value
*Acutalibacter muris*	0.000	1.355	0.010	0.000	0.133	0.000	0.045	0.000	0.212	0.025	0.702
*Akkermansia muciniphila*	0.000	5.261	0.012	0.000	1.507	0.000	0.022	0.000	4.251	0.000	0.066
*Anaerofustis stercorihominis*	0.000	0.015	0.000	0.000	0.010	0.000	0.434	0.000	0.005	0.000	0.047
*Anaerotaenia torta*	0.000	2.182	0.313	0.000	0.872	0.133	0.001	0.000	1.350	0.202	0.235
*Anaerotruncus colihominis*	0.000	0.059	0.000	0.000	0.020	0.000	0.172	0.000	0.133	0.000	0.025
*Bacteroides acidifaciens*	0.177	2.719	0.938	0.010	1.719	0.483	0.025	0.138	6.990	1.249	0.036
*Bacteroides sartorii*	0.000	0.655	0.106	0.000	0.227	0.034	0.309	0.000	1.665	0.212	0.009
*Bacteroides stercorirosoris*	0.000	0.015	0.000	0.000	0.000	0.000	0.025	0.000	0.049	0.000	0.132
*Bacteroides uniformis*	0.000	0.049	0.010	0.000	0.030	0.000	0.003	0.000	0.217	0.025	0.013
*Bacteroides vulgatus*	0.000	0.108	0.000	0.000	0.025	0.000	0.001	0.000	0.453	0.000	0.246
*Burkholderia lata/contaminans/metallica*	0.000	0.084	0.025	0.000	0.069	0.015	0.005	0.000	0.059	0.012	0.066
*Butyricimonas phoceensis*	0.000	0.000	0.000	0.000	0.020	0.000	0.064	0.000	0.025	0.000	0.002
*Butyricimonas virosa*	0.000	0.025	0.000	0.000	0.049	0.000	0.066	0.000	0.074	0.015	0.001
*Christensenella timonensis*	0.000	0.054	0.000	0.000	0.059	0.000	<0.001	0.000	0.015	0.000	0.052
*Clostridium scindens*	0.000	4.384	0.510	0.089	0.665	0.241	0.005	0.025	1.567	0.377	0.375
*Clostridium viride*	0.000	0.350	0.049	0.000	0.128	0.034	0.010	0.000	0.236	0.057	0.359
*Clostridium xylanolyticum*	0.059	8.286	1.271	0.000	1.025	0.089	0.001	0.000	2.453	0.325	0.112
*Desulfovibrio desulfuricans*	0.133	4.345	1.131	0.000	0.946	0.074	0.001	0.000	2.015	0.187	0.021
*Eisenbergiella massiliensis*	0.000	0.113	0.000	0.000	0.000	0.000	0.001	0.000	0.074	0.000	0.584
*Enterococcus faecalis*	0.000	0.025	0.000	0.000	0.054	0.000	0.038	0.000	0.044	0.000	0.486
*Flintibacter butyricus*	0.000	0.788	0.320	0.000	0.394	0.079	0.001	0.000	1.842	0.185	0.451
*Harryflintia acetispora*	0.000	0.128	0.015	0.000	0.148	0.030	0.406	0.000	0.438	0.039	0.021
*Ihubacter massiliensis*	0.000	0.325	0.069	0.000	0.212	0.039	0.010	0.000	0.158	0.034	0.161
*Jeotgalicoccus aerolatus/huakuii/nanhaiensis/halophilus*	0.000	0.163	0.000	0.000	0.000	0.000	0.001	0.000	0.000	0.000	0.005
*Kineothrix alysoides*	0.000	2.823	0.520	0.000	0.571	0.064	0.001	0.000	1.256	0.123	0.031
*Lachnoclostridium pacaense*	0.000	0.128	0.000	0.000	0.000	0.000	0.011	0.000	0.108	0.000	0.727
*Lactobacillus intestinalis*	0.000	2.094	0.000	0.079	29.006	0.616	0.022	0.000	3.995	0.286	0.685
*Lactobacillus johnsonii*	2.291	50.382	24.750	4.202	62.821	27.193	0.018	0.192	59.929	15.828	0.951
*Lactococcus garvieae/formosensis*	0.000	0.015	0.000	0.000	0.000	0.000	0.032	0.000	0.000	0.000	0.001
*Muricomes intestini*	0.000	0.025	0.000	0.000	0.000	0.000	0.025	0.000	0.015	0.000	0.486
*Mycoplasma muris*	0.000	0.054	0.000	0.000	0.039	0.000	0.256	0.000	0.000	0.000	0.002
*Neglecta timonensis*	0.000	1.094	0.224	0.000	0.320	0.020	0.001	0.000	0.897	0.094	0.931
*Paludicola psychrotolerans*	0.000	0.074	0.000	0.000	0.000	0.000	0.001	0.000	0.054	0.000	0.537
*Pseudoflavonifractor phocaeensis*	0.000	1.547	0.335	0.000	0.340	0.034	0.001	0.000	2.128	0.074	0.294
*Ralstonia pickettii*	0.000	0.025	0.000	0.000	0.000	0.000	0.006	0.000	0.000	0.000	0.092
*Roseburia faecis*	0.000	7.818	1.025	0.000	1.685	0.118	0.004	0.000	7.592	0.197	0.341
*Roseburia intestinalis*	0.000	0.113	0.000	0.000	0.039	0.000	0.030	0.000	0.034	0.000	0.011
*Ruminococcus champanellensis*	0.000	0.000	0.000	0.000	0.734	0.000	0.032	0.000	0.000	0.000	1.000
*Staphylococcus xylosus*	0.000	0.163	0.000	0.000	0.000	0.000	0.001	0.000	0.020	0.000	0.001
*Streptococcus acidominimus*	0.000	0.015	0.000	0.000	0.015	0.000	0.012	0.000	0.000	0.000	0.320
*Streptococcus danieliae*	0.000	0.138	0.020	0.000	0.079	0.005	0.081	0.000	0.034	0.000	0.006

Note: Comparisons between groups (Control vs LPS and Control vs VPA) were performed using the two-sided White’s non-parametric *t*-test.

## 4. Discussion

An effective animal model is a necessity for studies on ASD. Two chemicals that can be easily administered during pregnancy, LPS and VPA, were selected for intervention in ICR mice to construct ASD progeny mouse models.

The behavior features of offspring mice tend to be correlated in the same litter (homogeneity within the group), but remain independent in different litters (heterogeneity between groups). Considering litter effects, the data of rat offspring have a hierarchical structure (pregnant – fetus) and do not meet the assumption of independence in the generalized linear mode (GLM) [42]. Therefore, mixed effects models are often used to analyze data with such characteristics, which can measure the estimation of the intervention effect (fixed effect) and the variance of the random effect. In this study, random effects analysis revealed that the self-grooming time and frequency of the LPS group were affected by litter effects, and so were adjusted for further behavior analysis.

### 4.1 Why were Mice Offspring Selected Depending on Sex and Behavior?

ASD-like behaviors in mice are sex-dependent [41] and the incidence of ASD in males is significantly higher than in females [4, 43], so we first performed behavioral tests on male mice. In addition, gut microbiota composition is sex-specific [44], so the selection of male mice might reduce the effect of hormones on gut microbiota.

### 4.2 Evaluation of ASD Induced by Chemical Exposure during Pregnancy in Mouse Offspring

Exposure to various harmful factors during gestation may affect the growth and development of the embryo (fetus) and health after birth and may cause deformities or diseases. This is validated by our study, as the number of pregnancies that progressed normally up until delivery in the two ASD models varied, and the number of offspring in each litter differed greatly (**Supplementary Table 1**).

Social interaction defects, as well as repetitive and stereotyped behaviors, are core symptoms of individuals with ASD. Self-grooming behavior in rodents is very similar to the repetitive and stereotyped behaviors in ASD [45]. We therefore compared the results of the three-chamber sociability test and self-grooming among the two ASD mouse models to provide a better understanding of how the two models reflect ASD-like behaviors.

For social interaction defects, both models showed a significant reduction in social time relative to the Control group. Compared with the Control group, we observed a significant decrease in the social time of both the LPS and VPA groups, while there was no significant difference in social frequency despite a decrease. The order of performance of the two models in reflecting social interaction defects was therefore LPS>VPA (Fig. 2).

For repetitive and stereotyped behaviors, the LPS and VPA models showed significant increases in self-grooming frequency. The self-grooming time in the VPA model was significantly increased, while it was increased in the LPS group but this was not statistically significant. We also observed that the self-grooming time in the VPA group was significantly more than that in the LPS group. The order of performance of the two models in reflecting repetitive and stereotyped behaviors was therefore VPA>LPS (Fig. 1).

Indeed, both ASD models displayed ASD-like behavior to some extent, similar to previous studies. Exposure to LPS during pregnancy increases the frequency and time of self-grooming in offspring rats [23], whereas the duration of social interaction is significantly shorter compared with that in the control group, especially in male mice [22, 46]. Exposure of maternal mice to VPA during pregnancy resulted in the offspring showing a significant reduction in social time and a significant increase in repetitive behaviors (such as self-grooming) [47].

### 4.3 Characteristics of Gut Microbiota in ASD Mouse Models

Imbalances in gut microbiota are closely related to the occurrence and development of ASD [26]. Although gut microbiota is not a genetic factor, it may affect the health of offspring in a “genetic way” [48].

We analyzed the composition of the gut microbiota of 51 ASD and 24 control mice. The Shannon index of the LPS model was lower than that of the Control group, but it was higher in the VPA group, which indicates a decreased diversity of the intestinal microbiota of the two ASD models (Table 1).

The abundance of four phyla, 36 genera, and 27 species was significantly changed in the two ASD models. At the phylum level, the ratio of *Firmicutes*/*Bacteroidetes* (F/B) was higher in the LPS group than in the Control group (1.80 vs 1.51), whereas it was lower in the VPA group (1.11 vs 1.51). The F/B ratio is closely related to gastrointestinal symptoms in ASD patients [49], but is affected by diet and regional differences [50, 51]. The phyla *Proteobacteria*, *Candidatus Saccharibacteria*, and *Verrucomicrobia* were significantly decreased in the LPS model, whereas no significant differences were observed in the VPA model (Table 2).

At the genus level, *Butyricimonas*, a genus significantly increased in ASD children [52] and patients with constipation [53], was significantly enriched in the VPA model (Table 3).

The abundance of *Lactobacillus* was significantly increased in the LPS model (Table 3). In children with ASD, *Lactobacillus* has been reported to be significantly higher [54, 55] or lower than in normal children [56]. These contradictions may be related to the fact that the beneficial role of *Lactobacillus* is species and strain dependent, and ASD may be related to certain species or strains of *Lactobacillus* [54].

The relative abundance of *Mailhella* was significantly decreased in the LPS model (Table 3). *Mailhella *can reduce sulfate to produce H_2_S, and may cause the sulfate in the host to be consumed and the concentration of H_2_S to increase [57]. Increased concentration of H_2_S destroys the disulfide bonds of mucin, thereby, reducing the integrity of the intestinal mucosal barrier [58]. Additionally, a reduction in sulfate content in humans increases the risk of ASD in children [59]. The opposite changes in the abundance of *Mailhella* in different ASD models demonstrate the complexity of ASD, indicating that exposure to different chemicals during pregnancy may cause ASD differently.

A significant reduction in the abundance of *Roseburia *was observed in the LPS model (Table 3). The abundance of *Roseburia* in children with ASD is significantly lower than that in normal individuals [60]. Besides being the most important butyric-producing bacteria [61, 62], *Roseburia* also participates in the metabolism of tryptophan, glutamic acid, and other amino acids [63]. It can produce neurotransmitters with neuroactivity (such as 5-HT and GABA) that regulate intestine or brain function and contribute to the regulation of corresponding mental symptoms and abnormal behaviors [63].

At the species level, 44 species with significant differences were observed in the two ASD models. The abundance of *Akkermansia muciniphila* decreased in both ASD models; however, the difference was not statistically significant in the VPA group (Table 4). The relative abundance of *A. muciniphila* in children with ASD is low, and the decrease in *A. muciniphila* abundance may be related to changes in intestinal mucus barrier function in children with ASD [64]. We previously reported that the abundance of *A. muciniphila* in children with ASD is significantly lower than that in normal children [32]. Furthermore, fecal microbiota transplantation increases the abundance of *A. muciniphila*, thereby playing a vital role in improving cognitive dysfunction and social behavior defects [65]. Recent research shows that supplementation of *Akkermansia spp.* was able to improve the social deficits in a mouse model of ASD induced by VPA exposure [66]. Thus, reduced abundance of *A. muciniphila* could be used as a potential target for microbiota-based prevention and treatment of ASD.

*Kineothrix alysoides*, an anaerobic bacterium, can ferment various sugars except cellulose and xylan to SCFAs [67]. Among these, butyric acid is vital in maintaining intestinal ecological balance and in alleviating anxious behavior in mice [68]. The abundance of *K. alysoides* in the LPS and VPA models was significantly decreased (Table 4).

Exposure to different chemicals during pregnancy could have different effects on the gut microbiota composition in ASD model offspring, and the significantly changed taxa in each ASD model have been observed in ASD patients, proving their relationship with the occurrence and development of ASD. These results may provide new ideas for research, prevention, and treatment of ASD.

In the current study, there was one limitation in that we only examined male mice to better reflect the behavior of ASD. Indeed, studying both sexes is crucial for the translation of preclinical research [69]. Although in some studies sex is not the primary variable of interest [70], we intend to include both sexes in our follow-up studies.

## 5. Conclusions

Our conclusions are as follows: (1) Exposure to LPS or VPA during pregnancy can cause ASD-like behavior in offspring mice, with the VPA-induced ASD model exhibiting more repetitive and stereotyped behaviors. (2) Exposure to different chemicals during pregnancy was able to affect the gut microbiota in offspring mice differently, and several gut microbes could be related to the development of ASD. Our results provide a reference for future studies on the etiology and mechanism underlying the pathogenesis of ASD.

## Availability of Data and Materials

The data generated during the current study are available in the National Omics Data Encyclopedia (NODE) repository (https://www.biosino.org/node/), under accession number OEX014719 (16s rRNA).

## Supplementary Material

Supplementary material associated with this article can be found, in the online version, at https://doi.org/10.31083/AP38790.
