# Supplementary material for: Comparison of Gut Microbiota in Two Different Maternal Exposure Models of Autism Spectrum Disorder in Mice
Source: Alpha Psychiatry. 2025 Feb 28;26(1):38790. doi: 10.31083/AP38790 (PMC11916071; doi:10.31083/AP38790)
Supplement: Supplementary file 1 [file 2757-8038-26-1-38790-s1.docx]

Supplementary Table 1. Female and male offspring from each litter of pregnant mice in different treatment groups.

| **LPS** | | | **VPA** | | | **Control** | | |
| --- | --- | --- | --- | --- | --- | --- | --- | --- |
| **Litter** | **Male** | **Female** | **Litter** | **Male** | **Female** | **Litter** | **Male** | **Female** |
| L1 | 5 | 6 | L1 | 4 | 5 | C1 | 3 | 6 |
| L2 | 7 | 2 | L2 | 6 | 6 | C2 | 4 | 8 |
| L3 | 2 | 8 | L3 | 6 | 9 | C3 | 7 | 6 |
| L4 | 7 | 7 | L4 | 3 | 3 | C4 | 10 | 5 |
|  |  |  | L5 | 6 | 3 |  |  |  |
|  |  |  | L6 | 5 | 9 |  |  |  |

Note: The table lists the number of pregnant mice in each treatment group and summarizes the number of males and females of offspring mice in each litter.

Supplementary Table 2. Weight data of male and female offspring mice at different weeks after birth in each treatment group.

| **Group** | **3 weeks** | | | **4 weeks** | | | **5 weeks** | | | **6 weeks** | | |
| --- | --- | --- | --- | --- | --- | --- | --- | --- | --- | --- | --- | --- |
|  | **Male** | **Female** | ***p* value** | **Male** | **Female** | ***p* value** | **Male** | **Female** | ***p* value** | **Male** | **Female** | ***p* value** |
| **Control** | 15.19 ± 2.61 | 15.17 ± 2.28 | 0.975 | 27.67 ± 3.54 | 24.55 ± 1.81 | <0.001 | 34.52 ± 3.27 | 29.42 ± 2.11 | <0.001 | 39.18 ± 2.29 | 31.31 ± 1.80 | <0.001 |
| **LPS** | 16.01 ± 2.27 | 14.08 ± 1.99 | 0.005 | 29.13 ± 2.16 | 24.85 ± 1.16 | <0.001 | 35.73 ± 2.28 | 30.33 ± 2.44 | <0.001 | 38.91 ± 2.10 | 31.38 ± 2.38 | <0.001 |
| **VPA** | 16.31 ± 3.16 | 15.92 ± 2.97 | 0.609 | 28.64 ± 3.32 | 25.53 ± 2.18 | <0.001 | 35.40 ± 3.10 | 30.08 ± 3.09 | <0.001 | 39.35 ± 3.29 | 32.30 ± 2.87 | <0.001 |

Note: ① The table shows the mean ± standard deviation body weight (g) data of male and female offspring mice in the three treatment groups at 3, 4, 5, and 6 weeks after birth. ② Comparisons between groups (Control vs LPS and Control vs VPA) were performed using Welch's *t*-test.

Supplementary Table 3. Statistical analysis of litter effects in each group (*p* value).

| **Group** | **Self-Grooming** | | **Three Chamber** | |
| --- | --- | --- | --- | --- |
|  | **Time** | **Frequency** | **Time Social** | **Time Non-Social** |
| **LPS** **vs** **Control** | 0.013 | 0.022 | 0.663 | 0.526 |
| **VPA** **vs** **Control** | 0.591 | 1.000 | 0.703 | 0.122 |

Note: ① The data shown in the table are the *p* values of litter effects. ② The random effects of behavioral indicators in each group were analyzed. Comparisons between groups (Control vs LPS and Control vs VPA) were performed using the Log Likelihood Ratio Test (LRT). The litter effects were considered to be significant when *p* < 0.05.

Supplementary Table 4. Intraclass correlation coefficients.

|  | **Unadjusted ICC** | **Adjusted ICC** |
| --- | --- | --- |
| **Self-grooming time** |  |  |
| LPS vs Control | 0.403 | 0.479 |
| VPA vs Control | 0.048 | 0.061 |
| **Self-grooming frequency** |  |  |
| LPS vs Control | 0.244 | 0.353 |
| VPA vs Control | / | / |
| **Time in social chamber** |  |  |
| LPS vs Control | 0.043 | 0.052 |
| VPA vs Control | 0.034 | 0.039 |
| **Time in non-social chamber** |  |  |
| LPS vs Control | 0.056 | 0.056 |
| VPA vs Control | 0.139 | 0.142 |

Note: The intraclass correlation coefficient (ICC) is the ratio of between-group variance to total variance; the higher the value, the greater the degree of aggregation.

Supplementary Table 5. Statistical analysis of fixed effects in each group.

| **Group** | **Self-Grooming** | |
| --- | --- | --- |
|  | **Time** | **Frequency** |
| **LPS** **vs** **Control** |  |  |
| Intercept | 27.502 | 2.665 |
| treat = LPS | 33.750 | 3.872 |
| F | 2.475 | 7.237 |
| df | 4.675 | 5.079 |
| *p* value | 0.181 | 0.043 |
| **VPA** **vs** **Control** |  |  |
| Intercept | 27.990 | 2.708 |
| treat = VPA | 50.684 | 3.058 |
| F | 10.434 | 16.363 |
| df | 6.258 | 52 |
| *p* value | 0.017 | <0.001 |

Note: The table summarizes the results of the fixed effects part of the mixed effects models. By using the type III analysis of variance with Satterthwaite's method to compare between groups (Control vs LPS and Control vs VPA), the corresponding F, df, and *p* values were obtained. The intercept represents the mean value of the control group and the coefficient represents the difference between the two treatment groups.
